# The harmonic organization of auditory cortex

**DOI:** 10.3389/fnsys.2013.00114

**Published:** 2013-12-17

**Authors:** Xiaoqin Wang

**Affiliations:** ^1^Department of Biomedical Engineering, Johns Hopkins University School of MedicineBaltimore, MD, USA; ^2^Tsinghua-Johns Hopkins Joint Center for Biomedical Engineering Research and Department of Biomedical Engineering, Tsinghua UniversityBeijing, China

**Keywords:** harmonicity, auditory cortex, marmoset, pitch, music

## Abstract

A fundamental structure of sounds encountered in the natural environment is the harmonicity. Harmonicity is an essential component of music found in all cultures. It is also a unique feature of vocal communication sounds such as human speech and animal vocalizations. Harmonics in sounds are produced by a variety of acoustic generators and reflectors in the natural environment, including vocal apparatuses of humans and animal species as well as music instruments of many types. We live in an acoustic world full of harmonicity. Given the widespread existence of the harmonicity in many aspects of the hearing environment, it is natural to expect that it be reflected in the evolution and development of the auditory systems of both humans and animals, in particular the auditory cortex. Recent neuroimaging and neurophysiology experiments have identified regions of non-primary auditory cortex in humans and non-human primates that have selective responses to harmonic pitches. Accumulating evidence has also shown that neurons in many regions of the auditory cortex exhibit characteristic responses to harmonically related frequencies beyond the range of pitch. Together, these findings suggest that a fundamental organizational principle of auditory cortex is based on the harmonicity. Such an organization likely plays an important role in music processing by the brain. It may also form the basis of the preference for particular classes of music and voice sounds.

## Harmonics in the hearing environment

A harmonic is a component frequency of a sound that is an integer multiple of a fundamental frequency. For example, if the fundamental frequency is *f*_0_, the harmonics have frequencies 2*f*_0_, 3*f*_0_, 4*f*_0_,…etc., each of which is periodic at *f*_0_. Furthermore, the sum of the harmonics is also periodic at *f*_0_. Spectrally, harmonic frequencies are equally spaced by the width of *f*_0_. Harmonics are essential components of music. They are produced by music instruments of many types whose designs result in resonances at harmonic frequencies (Campbell et al., 2006). For example, vibrating a string in a violin by a bow or finger produces harmonic sounds; playing one or more keys on a piano produces sounds that are rich in harmonics. Music played by instruments contains main intervals that can be expressed by small-integer ratios, such as 1:1 (unison), 2:1 (octave), 3:2 (perfect fifth), etc., which sound consonant to most people. The notion of “harmonicity” in this paper refers broadly to both harmonics that are integer multiples of a fundamental frequency and intervals defined by small-integer ratios. Harmonics are also unique features of human voices and animal vocalizations. Vocal apparatuses of many species, ranging from avian to rodents and primates, produce harmonic sounds. For example, when the air passes through the vocal folds of a human, the vocal folds oscillate at a fundamental frequency (or pitch). In the natural environment, harmonics are produced as a result of non-linear characteristics of acoustic generators or reflectors. We therefore live in an acoustic world full of harmonics. The perception of harmonics is not only essential for the understanding and appreciation of music, but also crucial for the auditory system to discriminate between vocal communication signals and environmental sounds (e.g., sounds from blowing wind, running water and waving trees). An important distinction of environmental sounds is that they are generally inharmonic whereas most vocalizations contain harmonic structures.

In addition to harmonics encountered in the acoustic environment, the auditory system also produces harmonics internally (Pickles, 1988). The cochlea generates non-linear distortion products that contain harmonics of frequencies heard (Robles et al., 1991). The non-linear processing in auditory nerve, cochlear nucleus and other structures leading to auditory cortex could also generate harmonic by-products. Therefore, during the early developmental period, the auditory cortex is flooded with both exogenous harmonics from the acoustic environment and endogenous harmonics generated within the auditory system. Given what we know about the developmental plasticity, the auditory cortex must be imprinted with harmonic structures. Such an “imprinting processing” is likely reinforced throughout evolutional history of a species. Cortical neural circuitry thus may have evolved to accommodate the hearing environment with the prevalence of harmonics. Subcortical structures of the auditory system may also have evolved to reflect harmonics in the hearing environment. One would expect that a fundamental organizational principle of auditory cortex be based on harmonic structures of sounds, which bears important implications for understanding how the brain processes music.

## Harmonically related frequency inputs to auditory cortex

A hallmark of neurons throughout the ascending auditory systems is the frequency tuning, measured by pure tone stimulation, which is first established in the cochlea (von Békésy, 1960). A typical auditory neuron is tuned (with excitatory responses) to one particular frequency, referred as the characteristic frequency (CF) or best frequency (BF), within the hearing range of a species. In the auditory nerve, a fiber is only tuned to a single frequency (Kiang et al., 1967). Beginning from the cochlear nucleus, some neurons are found to show a secondary frequency tuning in addition to CF (Marsh et al., 2006). The prevalence of the neurons tuned to more than one frequency increases along the ascending auditory pathway. We consider a neuron having a “multi-peak frequency tuning” if it shows frequency selectivity to more than one frequency when tested by pure tones. Such neurons usually are tuned to one frequency most sensitively (referred to as the CF) and may also be tuned to a second, third or forth discrete frequency. Cautions need be taken to ensure that tunings to secondary frequencies are not caused by non-linear harmonic distortions of the acoustic system used in the measurement. Multi-peak frequency tuning has been observed in various subcortical auditory structures such as the inferior colliculus (Portfors and Wenstrup, 2002) and in the auditory sector of the reticular nucleus (Villa, 1990).

In auditory cortex, while the majority of neurons appear to be tuned to a single frequency, a significant proportion of neurons are found to have multi-peak frequency tuning. Multi-peak cortical neurons are found in various mammalian species, from bats (Suga et al., 1983; Fitzpatrick et al., 1993), cats (Abeles and Goldstein, 1970; Phillips and Irvine, 1981; Sutter and Schreiner, 1991) to non-human primates (marmoset: Aitkin and Park, 1993; Kadia and Wang, 2003; Sadagopan and Wang, 2009; macaques: Rauschecker et al., 1997). When a neuron is tuned to more than one frequency, the relationship between these frequencies is sometimes found to be harmonic. In echo-locating bats, combination-sensitive neurons outside the primary auditory cortex (A1) usually show multiple excitatory peaks at harmonics of CF (e.g., 2CF, 3CF, 4CF, etc.), corresponding to spectral components of ultrasonic calls emitted by the bats that are harmonically related (Suga et al., 1983). Inside A1, some neurons show multi-peak frequency tuning that are not at harmonics of CF (Kanwal et al., 1999).

**Figure 1:**
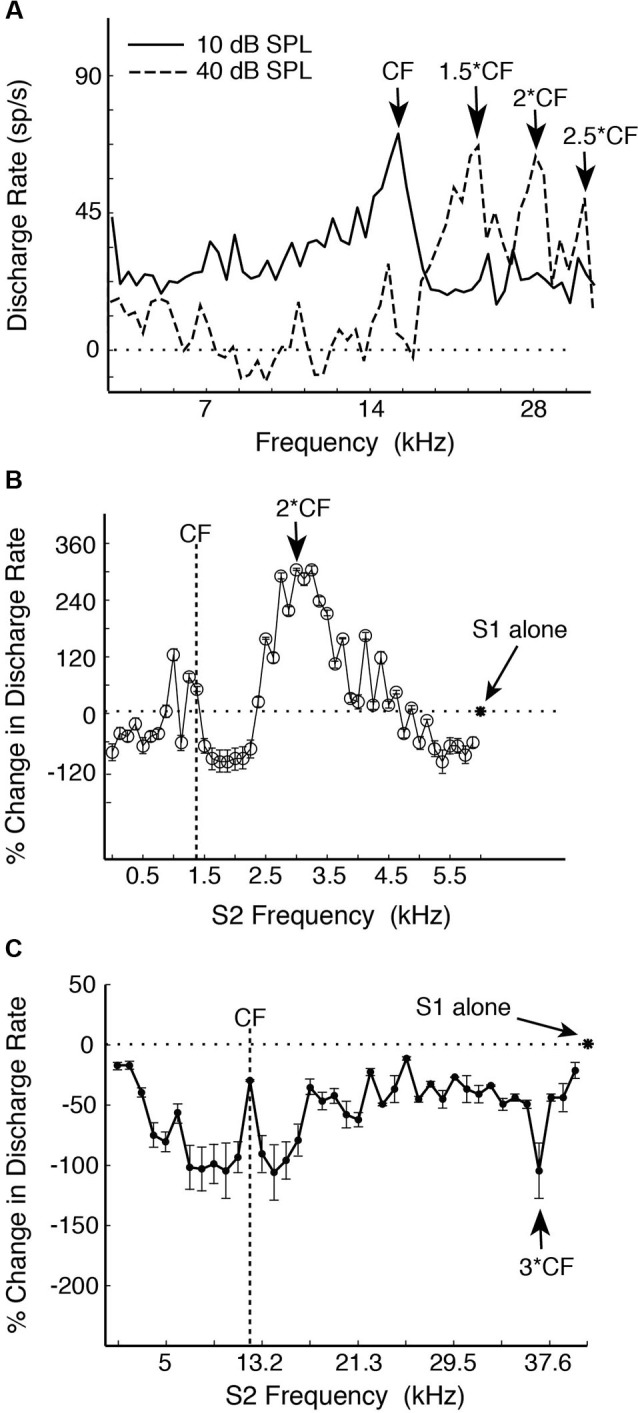
**Examples of single-peak and multi-peak neurons recorded in marmoset A1 (adapted from Kadia and Wang, 2003). (A)** Example of a multi-peaked A1 neuron with different excitatory frequency peaks at different sound levels revealed by single-tone stimulation. Discharge rate is plotted vs. tone frequency for two sound levels (10, 40 dB SPL). Several frequency peaks were identified. Note that the frequency peaks at both sound levels were harmonically related to the neuron’s CF of 14.5 kHz. **(B)** An example of two-tone facilitation in a single-peak A1 neuron. A two-tone pair is defined by Stimulus 1 (S1, fixed tone) and Stimulus 2 (S2, variable tone). S1 is at CF (1.47 kHz), 50 dB SPL and S2 at 0.12–5.88 kHz, 70 dB SPL (in linear steps of 122 Hz). This neuron has a monotonic rate-level function at CF with the threshold (Th) at 0 dB SPL. Percent change in discharge rate is plotted vs. S2 frequency. The asterisk and horizontal dotted line indicate the discharge rate of responses to the S1 tone alone. **(C)** An example of a single-peak A1 neuron showing distant off-CF inhibitions. This neuron has a non-monotonic rate-level function with a preferred sound level of 50 dB and Th of 20 dB. S1 is at CF (12.25 kHz), 50 dB SPL and S2 at 1–39.6 kHz, 40 dB SPL (in linear steps of 1000 Hz). The strongest distant off-CF inhibition is at 3CF (indicated by an arrow). The asterisk and horizontal dotted line indicate the discharge rate of responses to the S1 tone alone.

**Figure 2:**
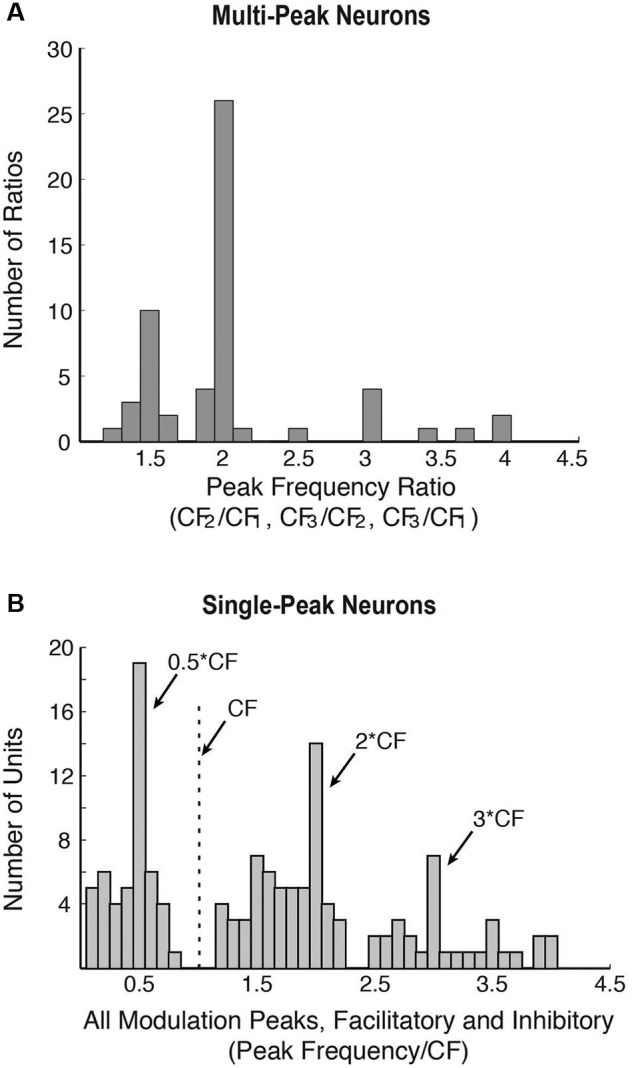
**Population properties showing harmonic interactions in marmoset A1 (adapted from Kadia and Wang, 2003). (A)** Distribution of all peak frequency ratios (CF_2_/CF_1_, CF_3_/CF_1_, and CF_3_/CF_2_) of multi-peak neurons. CF_1_, CF_2_ or CF_3_ are the frequency peaks of multi-peak neurons. Since a multi-peak neuron could have more than two peaks, it may be represented by more than one peak frequency ratio in this plot. A total of 56 peak frequency ratios were obtained from 38 multi-peak neurons (from Kadia and Wang, 2003). **(B)** Response modulations in the population of single-peak neurons in marmoset A1. Distribution of all modulatory peaks (facilitatory or inhibitory) measured from 76 of 113 neurons that showed facilitation and/or inhibition in their two-tone responses. There were a total of 139 measured peaks.

In the common marmoset (*Callithrix jacchus*)—a highly vocal primate species (Wang, 2000), about 20% of single neurons sampled from A1 under awake condition were found to have multi-peak frequency tuning (Kadia and Wang, 2003). Figure 1A show an example of such a neuron. This neuron has one excitatory peak at 10 dB sound pressure level (SPL; CF = 14.5 kHz) and three excitatory peaks at 40 dB SPL (21.9, 28.1 and 35.3 kHz, corresponding approximately to 1.5CF, 2CF and 2.5CF, respectively). In contrast to echo-locating bats, multi-peak neurons in marmoset A1 show excitatory peaks not only at integer multiples of CF (such as 2CF, 3CF, etc.), but also at some integer ratios of CF (such as 1/2, 3/2, etc.) as illustrated by the example neuron in Figures 1A, 2A shows a population summary of the excitatory frequencies of multi-peak neurons in relationship to CF found in marmoset A1 (Kadia and Wang, 2003). The distribution shows a dominant peak at 2CF (2:1 peak frequency to CF ratio) as well as a prominent peak at 1.5CF (3:2 peak frequency to CF ratio). In some but not all cases, the frequencies of the excitatory peaks of these multi-peak neurons fall into the frequency range of marmoset vocalizations (4–16 kHz) (Agamaite, 1997; Agamaite and Wang, 1997; DiMattina and Wang, 2006; Pistorio et al., 2006). In many cases, harmonically related excitatory peaks are outside the vocalization range of marmosets (Kadia and Wang, 2003). Similar to echo-locating sounds emitted by mustache bats, spectral components of marmoset vocalizations typically contain multiples of a fundamental frequency constrained by the vocal apparatus which is between 4–8 kHz for marmosets (DiMattina and Wang, 2006) and 30 kHz for mustache bats (Suga, 1989).

In mammalian species other than echo-locating bats, the proportion of multi-peak neurons is relatively small (less than 20% of samples by some estimates (Sutter and Schreiner, 1991; Kadia and Wang, 2003)), of which only a portion shows harmonically related excitatory peaks. About 80% of A1 neurons in marmosets are considered “single-peak neurons” when tested with a single pure tone, that is, they exhibited frequency tuning to a CF like auditory nerves fibers. However, when these neurons were tested with two simultaneously presented tones (with one tone placed at a neuron’s CF), a significant proportion of single-peak neurons showed harmonically related facilitation or inhibition in their two-tone responses between two frequencies (Kadia and Wang, 2003), suggesting that they receive inputs at frequencies other than at their CFs. Figure 1B shows two-tone responses of a single-peak neuron in marmoset A1. In this case, in addition to a tone presented at the neuron’s CF (S1), the frequency of a simultaneously presented second tone (S2) was varied. As one can see from Figure 1B, the two-tone combination generated a much stronger facilitatory response when S2 frequency was near 2CF. Using the two-tone paradigm, we found that many single-peak neurons in marmoset A1 exhibited harmonically related facilitation (Kadia and Wang, 2003). The two-tone combination can also generate inhibitory responses in many marmoset A1 neurons, some of which were harmonically related, as shown by an example in Figure 1C. This neuron’s response was nearly completely suppressed when the S2 tone was placed at 3CF, far away from its CF of 12.25 kHz. We refer to this type of inhibition as “distant inhibition” because it is distinctly different from the sideband inhibition flanking the excitatory region near CF (Schreiner et al., 2000). What makes this distant inhibition interesting and important is that it is often harmonically related to CF. There are a larger proportion of single-peak neurons in marmoset A1 that show harmonically related distant inhibition than facilitation. Figure 2B shows the distribution of peak frequency ratios for both facilitatory and inhibitory modulations in two-tone responses in single-peak neurons recorded from marmoset A1. In contrast to multi-peak neurons (Figure 2A), we did not observe a strong preference for one-octave separation between two tones in facilitatory modulation in single-peak neurons, but found a clear preference in inhibitory modulation when the second tone was one octave higher or lower in frequency than the first tone (see Figure 10 of Kadia and Wang, 2003). The distribution in Figure 2B shows prominent peaks centered on 1/2CF, 2CF and 3CF, indicating widespread harmonically related inputs to A1. Distant or long-range inhibitory influences in A1 have also been observed in the auditory cortex of other non-primate species (e.g., bat: Kanwal et al., 1999; cat: Sutter et al., 1999; gerbil: Kurt et al., 2008; Moeller et al., 2010).

Harmonically sensitive neurons have been found in the two core auditory cortical fields of awake ferrets, A1 and anterior auditory field (AAF; Kalluri et al., 2008). Evoked potentials and multi-unit activity recorded from A1 of awake macaque monkeys show sensitivity to harmonic complex tones in particular cortical locations (Fishman et al., 1998, 2000, 2013). Brosch et al. (1999) observed maximal enhancement of multi-unit responses in A1 of anesthetized macaque monkeys when a pair of tones were delivered with a stimulus-onset-asynchrony of 120 ms and a frequency separation of about one octave. A similar observation was made in A1 of anesthetized cats where the maximally enhanced responses occurred when the first tone was above one octave below or above the subsequently delivered second tone (Brosch and Schreiner, 2000). Brosch et al. (2013) reported that, in the caudomedial field of anesthetized macaque monkey auditory cortex, the likelihood of synchronization between spontaneous firings of a pair of simultaneously recorded multi-unit clusters was highest when the best frequencies of the two clusters were about an octave apart. Using spectrally dense stimuli, Noreña et al. (2008) found octave-based over-representations of a particular set of frequencies (3, 5, 10, 20 kHz) in both multi-unit activity and local field potential recordings obtained from anesthetized cat A1. However, it remains unclear why cat A1 shows over-representations of these particular frequencies. It should be noted that such over-representations of specific octave-related frequencies reported by Noreña et al. (2008) represent a different property of A1 than the harmonic sensitivity exhibited by single neurons in above mentioned studies (e.g., Kadia and Wang, 2003) which are not limited to specific frequencies (see Figure 4 of Kadia and Wang, 2003). The only other example of octave-based over-representations of specific frequencies has been reported in echo-locating bats, which corresponds to spectral components of ultrasonic calls emitted by the bats that are harmonically related (Suga et al., 1983).

Neurons with harmonically related facilitatory frequency peaks are more optimally stimulated by specific combinations of spectral elements than by these components separately. Therefore, these neurons can function to extract harmonic components embedded in complex sounds as a unitary object. Kadia and Wang (2003) showed that multi-peak neurons with harmonically related multi-frequency tuning (20% of marmoset A1 neurons) exhibited non-linear two-tone facilitation when tested by simultaneously presenting two tones at two discrete frequencies that a neuron is tuned to (see Figures 5, 6 of Kadia and Wang, 2003). It is also conceivable that such neural mechanisms can facilitate detection of signals in noisy environments by binding harmonic spectral features of an acoustic object. The frequency-specificity of the distant inhibition in single-peaked neurons suggests that the distant off-CF inhibition may have a different functional role than the distant off-CF facilitation. It further suggests that single-peaked neurons, which represent the majority of A1 neurons, may process harmonically related spectral components in a different manner than multi-peak A1 neurons. Just as harmonics are useful in assembling components of a complex acoustic object into one entity, they can also introduce confusion regarding the identification of the fundamental frequency. The strong inhibitory influences by harmonically related frequencies might enhance the perception and identification of the fundamental frequency. The harmonically related inhibition could also serve as a mechanism to remove unwanted harmonic artifacts in natural environment. It is also conceivable that, by combining harmonically related facilitation and inhibition, the auditory cortex could determine whether a sound is harmonic, a function that is important for a wide range of auditory perception.

## Anatomical basis for harmonic processing in auditory cortex

The physiological observations such as those presented in Figures 1, 2 suggest that A1 neurons receive harmonically related inputs via particular anatomical connections. The specific nature of such anatomical connections remains largely unknown. There are several possibilities on how the harmonic selectivity of A1 neurons could be formed. First, the harmonically related inputs could be combined subcortically and A1 simply inherits its harmonic sensitivity from MGB. Second, A1 neurons may acquire the harmonic sensitivity or modify the harmonic sensitivity that it inherits from MGB by receiving harmonically related inputs from other A1 neurons via long-range intracortical connections. Third, harmonic sensitivity of A1 neurons may be formed or shaped by feedback connections from secondary cortical areas that are more specialized for processing harmonic sounds. It has long been known that there exist extensive long-range horizontal connections within the primary sensory cortex including A1 formed by the axons of pyramidal cells (Gilbert and Wiesel, 1979; McGuire et al., 1991; Gilbert, 1998; Wallace et al., 2002; Moeller et al., 2010). In the supragranular layers of cat A1, the horizontal connections were found to extend as long as a few millimeters and span up to two octaves in CFs (Reale et al., 1983; Matsubara and Phillips, 1988; Ojima et al., 1991; Wallace et al., 1991; Winer, 1992; Kadia et al., 1999). Kaur et al. (2004) showed that receptive fields in rat A1 based on subthreshold EPSP and local field potential were remarkably broad, often spanning up to five octaves. Using a pharmacological inactivation method (muscimol), they further showed that intracortical connections provide non-CF inputs and contribute to the broad receptive field of A1 neurons. Wallace et al. (1991) showed that in cat auditory cortex intracortical connections displayed a periodic pattern along the tonotopic axis. However, the functional nature of the long-range horizontal connections in A1 remains largely unclear. Such intracortical connections may possibly provide harmonically related inputs to A1 neurons that underlie harmonically related two-tone facilitation and inhibition as well as other types of harmonic sensitivity.

**Figure 3:**
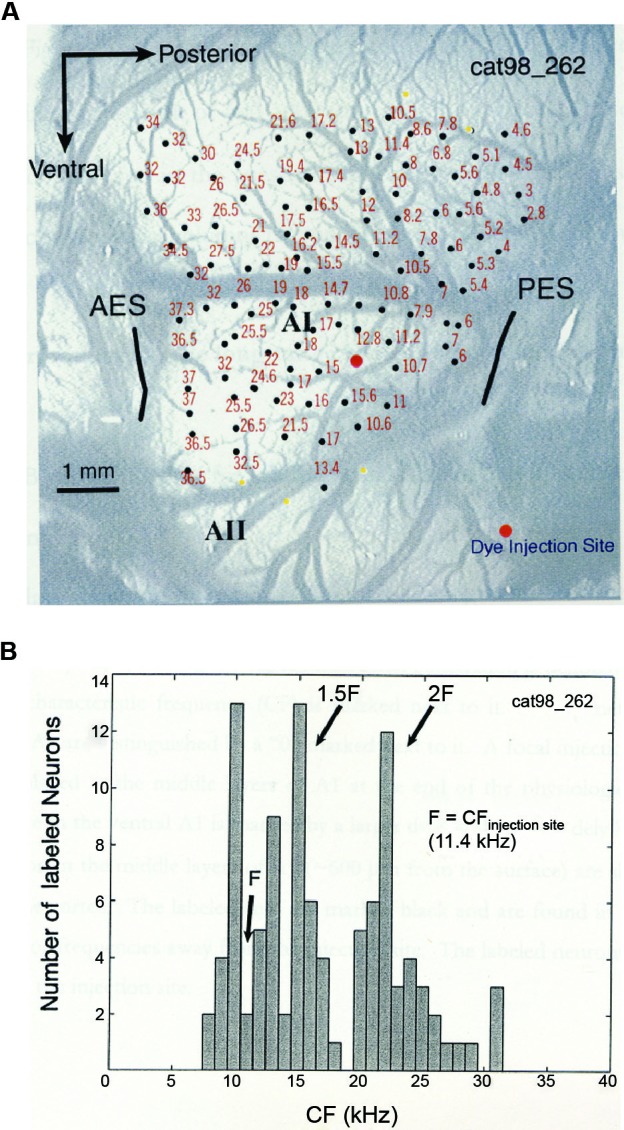
**Anatomic substrates for harmonic processing in cat A1 (adapted from Kadia et al., 1999). (A)** Tonotopic map of A1 obtained from a cat. Photograph of the left A1 is shown, overlaid with microelectrode penetration sites (dark circles). Numerical numbers next to each recording site indicate CF measured at that site. The large orange circle indicates the site of dye (anatomical tracer) injection. **(B)** Distribution of CFs of labeled neurons in one A1 horizontal section of the animal shown in **(A)**. CF of a labeled neuron was determined based on its location on the tonotopic map measured physiologically.

In a combined physiology and anatomy study of cat A1, it was found that anatomical tracers injected into a specific cortical location labeled neurons at other A1 locations that are harmonically related to the injection site (Kadia et al., 1999). Figure 3 shows data from this study. Figure 3A shows the tonotopic map of A1 obtained from a cat using the classic microelectrode mapping method. In cat A1, frequency tuning changes at a rate of approximately one octave per millimeter on the tonopotic map across cortical surface (Merzenich et al., 1975). After the mapping, an anatomic tracer was injected into a region of A1 tuned to about 11.4 kHz which resulted in labeled neurons both near and far away from the injection site. Figure 3B shows the distribution of the CFs corresponding to the cortical locations where labeled neurons were found. In addition to labeled neurons near the injection site, a larger concentration of labeled neurons was found at locations near 1.5CF and 2CF, which is suggestive of harmonically related long-range cortico-cortical horizontal connections in A1. Because horizontal connections originated from pyramidal cells are primarily excitatory (Gilbert and Wiesel, 1979; Martin and Whitteridge, 1984) and innervate both excitatory and inhibitory, GABAergic neurons (Kisvarday et al., 1986; McGuire et al., 1991), they may contribute to both facilitatory and inhibitory modulations between two distant frequencies. Although long-range horizontal connections have not yet been directly demonstrated in the auditory cortex of the common marmoset, their existence in other primate and mammalian species suggests strongly that this anatomic feature is preserved in the sensory cortex across many species. Future studies with more precise anatomical labeling techniques are required to investigate functional properties of such anatomical connections.

## Harmonic pitch processing by auditory cortex

Pitch is a fundamental building block of music and its processing by the brain is crucial for music perception, speech perception and auditory object recognition in a complex acoustic environment. Changes in pitch are used to convey information in both music and speech, especially in tonal languages (e.g., Chinese, Vietnamese and Thai). Pitch is closely associated with the perception of harmonically structured or periodic sounds. The perception of fundamental frequency of complex sounds with harmonic structures and its corresponding neural mechanisms in cortex is therefore of particular importance. Theoretically, pitch can be computed using either spectral or temporal acoustic features of a sound (Plack and Oxenham, 2005; Oxenham, 2012). Complex sounds containing components at integer multiples of a common fundamental frequency are spectrally periodic, which can theoretically be measured using a harmonic template (a form of spectral pattern matching) (Goldstein, 1973). A modeling study shows that such templates could emerge from the development of the auditory system (Shamma and Klein, 2000). Any harmonic template-based mechanism must be sensitive to both the frequency spacing between harmonics and the absolute frequency of each harmonic component. A harmonic template mechanism can only function with resolved harmonics. In humans, the first 5–8 harmonics of a complex tone are resolved (Plomp, 1964; Plomp and Mimpen, 1968; but see Bernstein and Oxenham, 2003). Resolvability in marmosets is different from that in humans, with only the first four harmonics resolved at low fundamental frequency (Osmanski et al., 2013). This difference is likely the result of a smaller marmoset cochlea than that of humans (Johnson et al., 2012).

**Figure 4:**
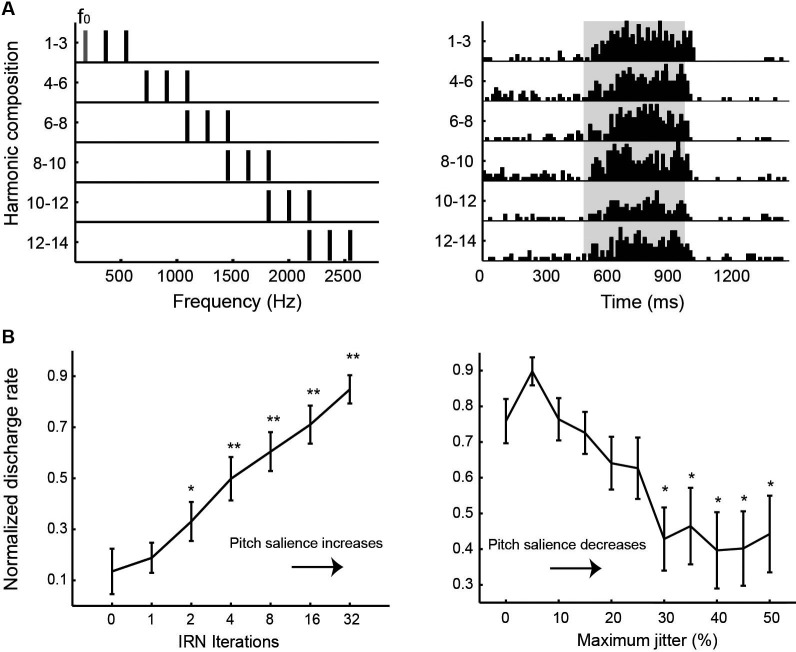
**Examples of pitch-selective responses recorded in marmoset auditory cortex (adapted from Bendor and Wang, 2005). (A)** *Left*: Frequency spectra of a series of harmonic complex stimuli. The fundamental frequency component (*f*_0_) and its higher harmonics have equal amplitudes of 50 dB SPL. *Right*: Peristimulus time histograms of a pitch-selective neuron’s responses to the harmonic complex stimuli. Stimuli were presented from 500 to 1000 ms (indicated by the shaded region on the plot). **(B)** Responses of pitch-selective neurons increases with increasing pitch salience. *Left*: Averaged population response of pitch-selective neurons as a function of the iterations of iterated rippled noise (IRN) stimuli. The response to IRN stimuli with zero iterations is used as a reference for statistical comparison at other iterations (* *p* < 0.05, ** *p* < 0.01). *Right*: Averaged population response of pitch-selective neurons to irregular click trains as a function of maximum jitter. The response to a regular click train is used as a reference for statistical comparison at other jitter values (* *p* < 0.05).

**Figure 5:**
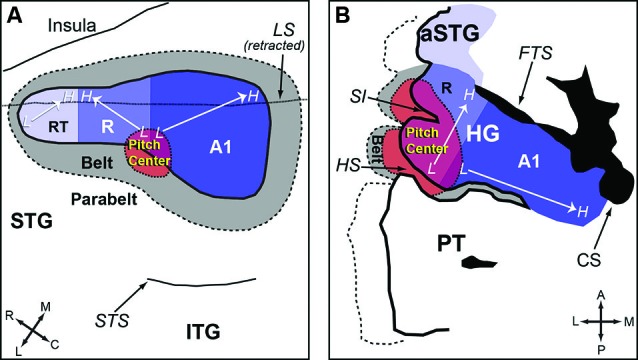
**Anatomic organization of auditory cortex and the location of the pitch-center (adapted from Bendor and Wang, 2006). (A)** An enlarged view of the superior temporal gyrus (STG) of marmoset, showing the core, belt, parabelt areas and the location of the pitch-center. The borders between each auditory area are estimated based on data from Bendor and Wang (2005) and Pistorio et al. (2004). **(B)** An enlarged view of HG in humans. A1 is presumed to occupy the medial portion of HG (with variability between subjects). The location of neighboring areas (R, pitch-center, lateral belt) is an approximation based on Schneider et al. (2005); Formisano et al. (2003); and Patterson et al. (2002). *Legend:* HG-Heschl’s gyrus, STG-Superior temporal gyrus, ITG-Inferior temporal gyrus, aSTG-Anterior superior temporal gyrus, PT-Planum temporale, SI-Intermediate sulcus, HS-Heschl’s sulcus, CS-Circular sulcus, FTS-First transverse sulcus, LS-Lateral sulcus, STS-Superior temporal sulcus, A1-Primary auditory cortex, R-Area R (rostral auditory cortex), RT-Area RT (rostrotemporal auditory cortex).

Auditory cortex of humans and non-human primates contains a “core” region, composing of A1, rostral area (R) and rostral-temporal area (RT). The core region is surrounded by “belt” and “para-belt” regions (Kaas and Hackett, 2000; Hackett et al., 2001). A pitch-center has been identified in non-primary auditory cortex of marmosets where neurons with pitch-selective responses were found (Bendor and Wang, 2005). These pitch-selective neurons not only were tuned to low frequency pure tones, but also responded to missing fundamental harmonic complex sounds with a pitch near a neuron’s CF. They did not, however, respond to individual components in a harmonic complex tone that were outside its tone-derived excitatory frequency response area. An example of a pitch-selective neuron is shown in Figure 4A. The location of pitch-selective neurons is in a low-frequency region of auditory cortex near the anterolateral border between A1 and R, similar to a pitch-region found in human auditory cortex (Figure 5; Bendor and Wang, 2006). A typical pitch-selective neuron also responded to an array of spectrally dissimilar pitch-evoking sounds (harmonic complex tones, click trains, iterated ripple noise) when the pitch was near the neuron’s preferred fundamental frequency. It was also found that pitch-selective neurons increased their firing rates as the pitch salience increased and preferred temporally regular sounds (Figure 4B), in agreement with the human imaging studies by Patterson et al. (2002) and Penagos et al. (2004). The anatomical studies of Brodmann (1909) and others suggested that the structure of the temporal lobe is largely preserved across primate species (New World monkeys, Old World monkeys and humans). Such a similarity suggests that the pitch-center identified in marmosets may exist in other non-human primate species as well and that this pitch-center shares similar functions as the lateral Heschl’s gyrus (HG) in humans where a pitch-selective region has been identified (Patterson et al., 2002; Penagos et al., 2004; Bendor and Wang, 2006).

It has been demonstrated that marmosets are able to behaviorally perceive pitch of harmonic complex and pitch is extracted by marmosets using temporal envelope cues for lower pitch sounds composed of higher-order harmonics, whereas spectral cues are used for higher pitch sounds with lower-order harmonics (Osmanski et al., 2013). Physiological experiments have further showed that pitch-selective neurons within the putative pitch-center of marmoset auditory cortex can use either temporal envelope or spectral cues in a harmonic complex for pitch extraction, depending on pitch values and harmonic compositions (Bendor et al., 2012). The major determinant of whether pitch is extracted using temporal envelope and/or spectral cues is the fundamental frequency and harmonic order of the complex tone. These findings indicate that two different mechanisms are used by the auditory system of the marmoset to extract pitch. The temporally based mechanism is sensitive to both the temporal regularity and repetition rate of the envelope of the acoustic signal. The spectrally based mechanism is sensitive to the harmonicity and fundamental frequency of a harmonic complex tone, but not to the envelope of the acoustic signal. The behavioral and neurophysiological studies of pitch processing in the marmoset support the dual-pitch processing mechanisms, originally proposed by psychophysicists based on human studies, whereby pitch is extracted using a combination of temporal envelope and spectral cues (Plack and Oxenham, 2005).

When considering neural mechanisms for harmonic pitch processing, it is important to differentiate between neurons that exact pitch embedded in harmonic complex and those that merely bear pitch information (Wang and Walker, 2012). This requires examining whether pitch is specifically represented by the neuron or cortical region under study, as well as determining that the physiological signal of the neurons correspond with the animal’s perception of pitch. Neural responses bearing pitch information or encoding acoustic parameters associated with pitch can be found throughout much of the ascending auditory pathway (e.g., Cariani and Delgutte, 1996a,b), though their specificity for pitch may increase at successive higher processing stages. Neural representations earlier in the system could serve as precursors to the neurons that ultimately compute pitch, but they may not represent the final stages of pitch processing.

Pitch processing by human auditory cortex has been investigated by a number of studies using various pitch-evoking stimuli. Patterson et al. (2002) used melodies composed of IRN stimuli in an fMRI imaging study and localized a “pitch center” to lateral HG and regions anterior to lateral HG in the right hemisphere. Penagos et al. (2004) used four harmonic complex sounds that had either a low or high pitch and occupied either a low or high spectral range to probe pitch processing by the human brain using fMRI imaging technique. They identified bilaterally a restricted region of non-primary auditory cortex in the lateral HG anterolateral to A1 that responded more strongly to stimuli with high pitch salience than those with low pitch salience. Furthermore, the activity in this region was not significantly different in the high pitch salience stimuli when comparing across different fundamental frequencies or frequency ranges. All together, a number of human imaging studies have confirmed the location of a pitch-center in lateral HG (Patterson et al., 2002; Gutschalk et al., 2004; Penagos et al., 2004; Hall et al., 2005, 2006; Ritter et al., 2005; Schneider et al., 2005; Chait et al., 2006; Hall and Plack, 2007, 2009; Schönwiesner and Zatorre, 2008; Puschmann et al., 2010).

Some studies have suggested that there might be more than one region in human auditory cortex for pitch processing (Hall and Plack, 2007, 2009). In Hall and Plack (2007) study the authors used a dichotic pitch stimulus known as the Huggins pitch, a weak pitch percept created by playing a broadband noise to each ear that are identical except for a narrow frequency band (190–210 Hz in this study) in which a phase shift is introduced in one of the ears relative to the other. The Huggins pitch stimulus was found to evoke a weak activation of cortex and no significant activity in lateral HG. Hall and Plack (2007) suggested that lateral HG may be only involved in processing diotic rather than diochotic pitch. However, a recent fMRI study by Puschmann et al. (2010) found pitch-related activation at the lateral end of HG in both hemispheres with dichotic pitch stimuli, providing further evidence for a general involvement of this region in pitch processing. It should be pointed out that interpretations of the human imaging studies depend critically on differences between responses to pitch and control stimuli other than the presence of pitch (Griffiths and Hall, 2012), which may explain differences in observations from experiments using different pitch-evoking or control stimuli. One desired property of a pitch processing region is that it should show pitch constancy, i.e., the same (or similar) response to a given pitch value and strength regardless of the particular associated stimulus. Hall and Plack (2009) attempted to address this issue by playing to the same listeners a variety of pitch-evoking stimuli but failed to show that all pitch is processed in a single locus in auditory cortex. Their results suggest that parts of the planum temporale (PT) are more relevant for pitch processing than lateral HG. In some listeners, pitch responses occurred elsewhere, such as the temporo-parieto-occipital junction or prefrontal cortex.

In summary, the majority of human imaging studies have pointed to a cortical region for pitch processing at the lateral end of HG anterolateral to A1 that mirrors the location of the pitch-center found in marmosets (Bendor and Wang, 2006; Griffiths and Hall, 2012). Whether this pitch-center is the sole pitch processing region in auditory cortex remains an open question. Because of technical limitations in single-unit recordings, not all auditory cortex areas in marmosets have been systematically studied for their possible pitch-processing functions. The notion of multiple pitch processing regions in auditory cortex, if it turns out to be true, would not be all that surprising given the complexity of pitch perception (Plack and Oxenham, 2005). Further studies need to investigate specific roles played by each cortical region that is involved in pitch processing (e.g., which aspects of pitch processing are processed by a specialized cortical region). Finally, anatomical basis for pitch processing in auditory cortex has yet been studied. It is conceivable that the long-range horizontal connections in auditory cortex discussed above play an important role in cortical processing of harmonic pitch.

## Temporal periodicity processing in auditory cortex

In addition to studies of harmonically related spectral inputs, a number of studies have also shown neural selectivity for temporal periodicity. Periodic sounds have a “regular” temporal structure as opposed to noises or random temporal sequences that have “irregular” temporal structures. The coding of periodic sounds in auditory cortex is often related to the neural processing of harmonics. Neural recordings in gerbils showed that A1 neurons could respond to the periodicity of amplitude-modulated tones with the spectral components located outside neuron’s excitatory frequency response area, but with the periodicity ranging much higher than pitch range found in humans (Schulze and Langner, 1997). Schulze et al. (2002) has also reported a semi-circularly shaped map of best fundamental frequency in gerbil auditory cortex using optical imaging techniques. Langner et al. (2002) reported a topographic arrangement of periodicity information in chinchilla auditory cortex that was orthogonal to the tono­topic axis.

Neurons that are preferentially driven by temporally modulated periodic sounds have been observed throughout auditory cortex in many species (Joris et al., 2004), including several non-human primates (Bieser and Muller-Preuss, 1996; Steinschneider et al., 1998; Liang et al., 2002; Malone et al., 2007). A large proportion of neurons in marmoset A1 showed preferential responses to amplitude- or frequency-modulated tones and, interestingly, some of these neurons could only be driven by temporally modulated tones but not by unmodulated pure tones (Liang et al., 2002). Neurons in marmoset auditory cortex are also found to be responsive to periodic click train stimuli, by either stimulus synchronized or unsynchronized discharges in both A1 (Lu et al., 2001) and rostral fields (Bendor and Wang, 2007, 2008).

However, the response to temporally modulated periodic sounds alone can not differentiate whether a neuron or cortical region responds to temporal regularity or average repetition rate. This issue was not examined in the studies referred to above. Using MEG imaging method, Gutschalk et al. (2002) identified two separate sources adjacent to A1 in humans that exhibit differential sensitivity to temporal regularity tested by click trains. One source, located in lateral HG, was particularly sensitive to regularity and largely insensitive to sound level. The second, located just posterior to the first in PT, was particularly sensitive to sound level and largely insensitive to regularity. In marmosets, except within the pitch-processing region, we find that neurons in auditory cortex respond to temporally varying acoustic signals with repetition rates in the range of pitch, yet they do not show sensitivity to temporal regularity, i.e., they have similar responses to periodic and aperiodic acoustic pulse trains with the same repetition rate (Bendor and Wang, 2010). In contrast, neurons within the pitch-processing region were found to be sensitive to temporal irregularity and decrease their firing rates for aperiodic acoustic signals.

## The harmonic organization hypothesis of auditory cortex

From the evidence reviewed in this article, we can conclude that harmonicity (spectrally) and periodicity (temporally) processing is a unique feature of auditory cortex. Harmonicity and periodicity are two closely related characteristics of sounds. A sound with harmonic structure spectrally usually has a periodic waveform temporally, although the periodicity depends on the phase relationships between the spectral components within the sound. Likewise, a periodic sound like a click train or a sequence of tone or noise pulses has harmonic components in its spectrum. Although a sinusoidal amplitude-modulated sound has only two sideband components besides the carrier component, its spectrum resembles a harmonic spectrum more than inharmonic ones. In a broader sense, neural coding of harmonicity and periodicity can be considered the coding of spectral or temporal regularity. From the information-coding standpoint, a spectrally or temporally regular sound can encode specific acoustic information, whereas a truly irregular sound (spectrally and temporally) like a white noise or random click train encodes little specific information other than parameters associated with its statistical structure. Given the mirror relationship between the harmonicity and periodicity, we may consider their neural representations being governed by a unified framework.

In A1, neurons show characteristic responses to harmonic spectral structures and periodic temporal modulations. In the pitch-region of non-primary auditory cortex in primates, pitch-selective neurons exhibit unique properties for processing harmonic pitch and temporal regularity that are not observed in A1. Much remains unknown on harmonic processing outsides A1 and the pitch-region. I propose here a hypothesis that *a fundamental organizational principle of auditory cortex is based on the harmonicity*. Under this hypothesis, neurons in A1 may be organized and inter-connected by their harmonic properties and various auditory cortical areas outside A1 may be specialized in processing particular harmonic or periodic structures of sounds. In this scenario, the pitch-center anterolateral to A1 is only one of several such cortical areas of a harmonics-based functional organization. Other cortical areas outside A1 may be specialized in processing, for examples, harmonic structures outside the pitch domain, spectral and temporal structures that contain preferentially used music intervals, etc. The diversity of harmonic interactions in A1 responses revealed by two-tone modulations (Figures 1, 2) suggests possible readouts by different higher auditory cortical areas. Recent imaging studies (Hall and Plack, 2007, 2009) demonstrating activations in multiple cortical areas in humans by different types of pitch evoking stimuli adds further support to the proposed harmonic organization of auditory cortical processing.

In summary, a harmonicity-based organizational principle of auditory cortex has profound implications for music processing by the brain. Future investigations will need to study physiological, anatomical and developmental bases of the hypothesized harmonic organization across mammalian species. There are a number of important questions to be asked. For example, what kinds of harmonic structures are processed by individual and populations of neurons in each auditory cortical area? How do neurons accomplish the computation to extract pitch and other harmonic structures from complex sounds? Anatomically, are neurons within a cortical area and between cortical areas connected via harmonically structured projections? How such anatomical projections are formed in the developing brain? Are they plastic and subject to modifications by an animal’s acoustic experience? Are there genetic codes to guide the development of the harmonic organization of auditory cortex? To answer these questions, researchers will need to employ techniques that enable the observation of population activity at the single neuron resolution (e.g., two-photon imaging) and methodologies that allow perturbations of neural networks (e.g., optogenetics). Another crucial question to ask is how the harmonic organization of auditory cortex underlies the perception of harmonic structures of sounds such as those found in music and vocalizations. Finally, one needs to know whether the harmonic organization proposed here is a universal principle across mammalian species and whether there are any species-specific variations from rodents to non-human primates and humans.

## Conflict of interest statement

The author declares that the research was conducted in the absence of any commercial or financial relationships that could be construed as a potential conflict of interest.
